# High- and Low-Complexity Features of Non-Critical Adult Patients in the Emergency Department

**DOI:** 10.3390/jcm15031280

**Published:** 2026-02-05

**Authors:** Andrea Fabbri, Laura Pistore, Flavio Bertini, Barbara Benazzi, Danilo Montesi

**Affiliations:** 1 Emergency Department, Local Health Agency of Romagna, Presidio Ospedaliero Morgagni-Pierantoni, Via C Forlanini 34, 47121 Forlì, Italy; bbenazzi1@gmail.com; 2 Department of Computer Science and Engineering, University of Bologna, Mura Anteo Zamboni 7, 40126 Bologna, Italy; laura.pistore@smartdata.unibo.it (L.P.); danilo.montesi@unibo.it (D.M.); 3 Department of Mathematical, Physical and Computer Sciences, University of Parma, Parco Area delle Scienze 53/A, 43124 Parma, Italy; flavio.bertini@unipr.it

**Keywords:** main complaint, non-critical, characteristics, clinical diagnostic workload

## Abstract

**Background/Objectives**: In the Emergency Department (ED), non-critical patients are classified as Triage Level (TL) 3 on arrival if they are assessed as having a high-level of complexity (HLC), or as TL4–5 if they are assessed as having a low-to-mild level of complexity (LLC). These levels are based on the estimated resources needed. This study aimed to identify the characteristics associated with an HLC or LLC by considering a group of variables from the presentation profile (PP) and clinical diagnostic workload (CDW), assessing ex post whether the assignment of complexity levels based on a priori estimation of the number of resources needed can be considered adequate. **Materials and Methods**: This retrospective multicentre study involved four first-level EDs and included patients between 2023 and 2024. **Outcome Measures**: The variables tested in a logistic model were those of the PP (age, sex, chief complaint, National Early Warning Score (NEWS), Numeric Rating Scale (NRS) and those of the CDW (diagnostic tests, interventions and therapy, assistance, and ED length of stay). **Results**: Of the 335,507 subjects considered, the average age was 59 years (interquartile range [IQR], 25), with 43.3% of cases classified as TL3. An NRS ≥ 7, ECG, urgent laboratory tests, NEWS > 6, need for a stretcher, and male gender were associated with TL3, whereas obstetric–gynaecological complaints, environmental complaints, skin-presenting complaints, and intramuscular therapy were associated with TL4–5. **Conclusions**: In non-critical patients a defined group of features were associated with different levels of complexity, going beyond the standard criterion based on the resources needed. These results could help clinicians improve the appropriateness of ED care pathways.

## 1. Introduction

### 1.1. Background/Rationale

The ED under study utilises the Canadian Triage and Acuity Scale (CTAS), a five-level triage and acuity system that categorises patients by urgency and establishes maximum waiting time targets for initial medical assessment. On this scale, cases defined as ‘critical’ are considered those at TL1 (resuscitation) and TL2 (emergency), while those at TL3 (emergency), TL4 (minor emergency) and TL5 (non-emergency) are considered ‘non-critical’ [1].

Patients defined as non-critical upon arrival usually do not require immediate or urgent life-saving interventions. However, they may present with multiple or severe chronic conditions, atypical or non-specific symptoms, psychosocial problems or functional limitations. These factors can sometimes make assessment and final diagnosis very difficult [2]. These cases are classified upon arrival on the estimated resource needed to complete the clinical diagnostic pathway [3].

High-complexity-level cases (HLC) are those with a high resource demand, while low-level-of-complexity cases (LLC) typically present with a single, clearly defined, minor, or self-limiting problem. The high complexity of Level 3 stems from a complex decision-making process, which considers, for example, pain intensity, age, or comorbidities compared to the primary complaints. Studies highlight the risk of selection bias, especially in crowded settings [4]. Examples of an LLC include a simple minor lesion, an uncomplicated localised infection, or a new medication administration, with a diagnosis that can usually be identified from a limited number of possibilities [5,6].

Under-triage and mis-triage have been associated with an unfavourable outcome [7], whereas over-triage has been linked to overcrowding and unnecessary hospital admissions [8].

### 1.2. Objectives

This study aimed to identify the characteristics according to the two groups of PP and CDW variables that define an HLC in subjects classified as TL3 and those that define an LLC in subjects classified as TL4–5, thereby assessing ex post whether the assignment of complexity levels based on a priori estimation of pathways can be considered adequate. These findings could inform strategies to improve the appropriateness of ED care pathways.

## 2. Materials and Methods

### 2.1. Participants

This is a multicentre and retrospective analysis from digital health records collected from the official registry of the Local Health Agency of Romagna, Italy, between 1 June 2023 and 31 December 2024 (18 consecutive months), including 495,119 encounters/1,172,853 inhabitants, from four 1st-level EDs.

**Figure 1:**
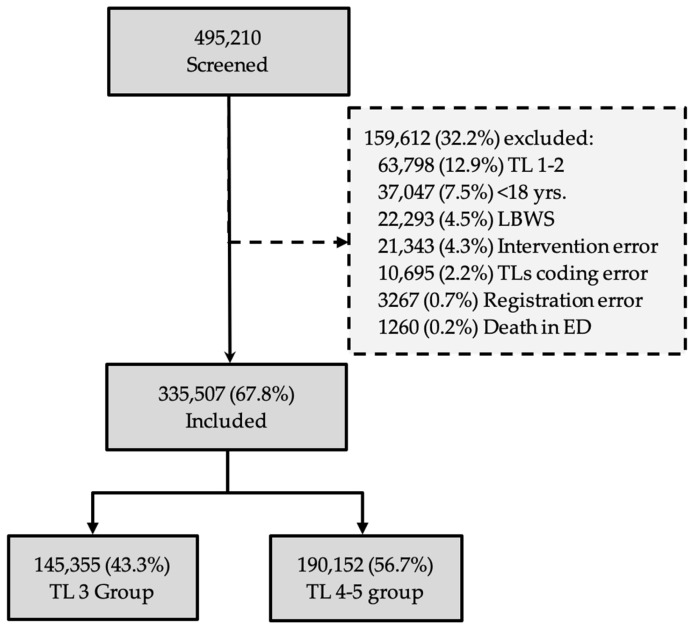
The flow diagram of the study.

A total of 495,210 consecutive patients registered upon arrival at triage were considered (Figure 1). A total of 159,703 subjects (32.2%) were excluded from the analysis for the following reasons: 63,798 (12.9%) due to critical conditions (TL1-2); 37,047 (7.5%) aged < 18 years; 22,293 (4.5%) because they left the ED without being seen; 21,343 (4.3%) due to intervention errors, e.g., those who left the ED during treatment (LDT); 10,695 (2.2%) cases due to coding errors; 3267 (0.7%) due to registration errors; 1260 (0.2%) due to death during ED stay. Following the selection process, 335,507 cases (67.8% of the total) were analysed comparing TL3 and TL4–5.

### 2.2. Variables

The PP of patients using the summary statistic was reported in Table 1. The patients’ characteristics included sex and age (by decades) and their NEWS to provide an objective, vital-sign-based assessment that detects early clinical deterioration [9]. In the analyses, the NEWS was reported as a categorical variable (0–4 low risk, 5–6 medium risk, >6 high risk). In the PP, the severity of pain was always included, according to the numerical rating scale (NRS) considered as different categories: 0–3 mild, 4–7 moderate, >7 severe [10]. Incorporating the NRS improves triage levels, ensuring that patients experiencing severe pain are given a higher priority, while reducing reliance on nurses’ judgement alone [11].

The main complaint for the visit was considered using the Canadian Emergency Department Information System (CEDIS) Complaints List (V2.0) classification [12]. All presenting complaints were further divided into two categories (Table 2), traumatic vs. non-traumatic origin.

In order to estimate the CDW, the following were considered as estimated resources required to complete diagnosis and care planning: number of laboratory tests, interventions, and therapy beyond the medical visit. Diagnostic tests and interventions were considered: radiologic imaging (e.g., X-rays), laboratory tests (e.g., blood tests), rapid tests (swab), procedures such as sutures or urinary catheterization, intravenous (IV) or intramuscular (IM) medications (but not oral medications), consultation with specialists, simple or complex interventions (e.g., mild sedation, dressings), intravenous therapy. The following were excluded from the intervention series: oral medications, simple wound care, crutches/splints, prescriptions, and basic anamnesis or physical examination.

The level of assistance was categorised into three levels: ambulatory patients (low-level), wheelchair users (medium-level), and stretcher patients (high-level), with the latter requiring a high CDW. Estimated EDLoS, assuming that a longer stay would require more resources, was also considered in the predictive model for the calculation of the CDW. The time was calculated as the difference between arrival at the ER and the end of the ED pathway (discharge or hospitalisation) and considered as both a continuous and categorical variable (<3 h, 3–6 h, 7–9 h and >9 h).

### 2.3. Statistical Methods

Data analysis was conducted on a cohort of 335,507 patients. Continuous variables were reported as either the mean (standard deviation, SD) or median [Interquartile range, IQR]. Differences in patient characteristics, along with the corresponding 95% confidence intervals (CIs), were calculated. A *p*-value of less than 0.05 was considered statistically significant for all analyses. Categorical variables were summarised as counts and percentages.

The NEWS and NRS were considered as continuous and categorical variables.

To account for differences among the subjects, age was divided into decades.

A generalised linear logistic model was developed. Selected variables were determined using the stepwise regression technique. Any multicollinearity arising from procedures in the ED was assessed and considered negligible for the aim of the study, as testing variable selections with alternative methods (e.g., Lasso and Elastic Net) resulted in a larger set of variables in which those exhibiting multicollinearity were still retained. Results were presented as odds ratios (OR) with two-sided 95% confidence intervals (95% CI).

An outcome risk score was computed for each patient based on the coefficients derived from the logistic regression model. The accuracy of this risk score was evaluated by the area under the receiver operating characteristic (ROC) curve. The optimal cutoff point, i.e., the value that maximises both sensitivity and specificity, was determined using the Youden index [13].

To avoid ambiguity, no synthetic data were generated to handle missing data; thus, the analysis relied solely on complete cases. The statistical model was subsequently validated using event–number balancing techniques [14].

Analyses were performed using the Python programming language (version 3.10.12) in Jupyter Notebook. Key libraries included stats models (version 0.13.5), scipy (version 1.10.0), and scikit-learn (version 1.6.0).

## 3. Results

### 3.1. Descriptive Data

Table 1 shows the PP variable for the two groups. Males accounted for 50.2% of cases, more represented in the TL4–5 group (51.1%) than in the TL3 group (49.1%). The mean age of the population was 59 years (interquartile range [IQR], 25), higher in the TL3 group than in the TL4–5 group. A comparison between the decades shows a higher prevalence of young people in the TL4–5 group and, conversely, of subjects in the >80 age decades in the TL3 group.

Figure S1 (Supplementary) shows the gender distribution across decades in the TL3 and TL4–5 groups. Males in the TL4–5 group were most common in the 40–60 age group, while in the TL3 group they were most common in the 60–80 age group. In the TL3 group, there was a peak incidence of females in the 80–90 age group.

Subjects with a medium (5–6) and high (>6) NEWS risk score were more represented in the TL3 group, while those at low risk (0–3), as expected, were more represented in the TL4–5 group. In most cases (85%), pain was mild (NRS 0–3). However, more cases were moderate (NRS 4–7) and severe (>NRS 7) in the TL3 group (Table 1).

Table 2 compares chief complaint categories between the two groups, ranked by importance. The main differences between the two groups were in order of importance: environmental, mental health, cardiovascular, cerebral vascular, and the trauma category, which were more represented in the TL3 than in the TL4–5 group.

Table S1 (Supplementary) shows the type and number (%) of resources required for the diagnostic tests and procedures performed on the study population. The most common procedures are blood sampling, ECG, cannulation, swabs, specialist consultation, and urine test strips.

Table S2 (Supplementary) lists the number and frequency of tests and procedures performed in the two groups. Considering the full database, 31.6% of cases did not undergo any tests or interventions, 23.2% underwent one test or intervention, and 22% underwent two or more tests or interventions. A comparison between the two groups shows that TL3 cases require more resources.

Seventy-seven per cent of patients were in an upright position, 5.3% were in a wheelchair, and 17.7% were on a stretcher. An EDLoS < 3 h was more common in the TL4–5 group (35.8%) than in the TL3 group (19.6%; odds ratio (OR) 1.04; 95% confidence interval (CI) 1.03–1.06; *p* < 0.001). On the other hand, an EDLoS > 3 h was more likely in the TL3 group. Specifically, in the 3–6 h group (39.0% vs. 36.0%) (OR 1.10 95% CI 1.09–1.12, *p* < 0.001), 6–9 h (23.0% vs. 17.8%); (OR 0.96: 95% CI 0.94–0.97: *p* < 0.001), >9 h (18.4% vs. 10.4%) (OR 1.03 95% CI 1.01–1.05: *p* < 0.001).

### 3.2. Outcome Data

Stepwise feature selection of the PP and CDW groups using a logistic model on randomly under-sampled data revealed a set of variables that entered the model.

**Figure 2:**
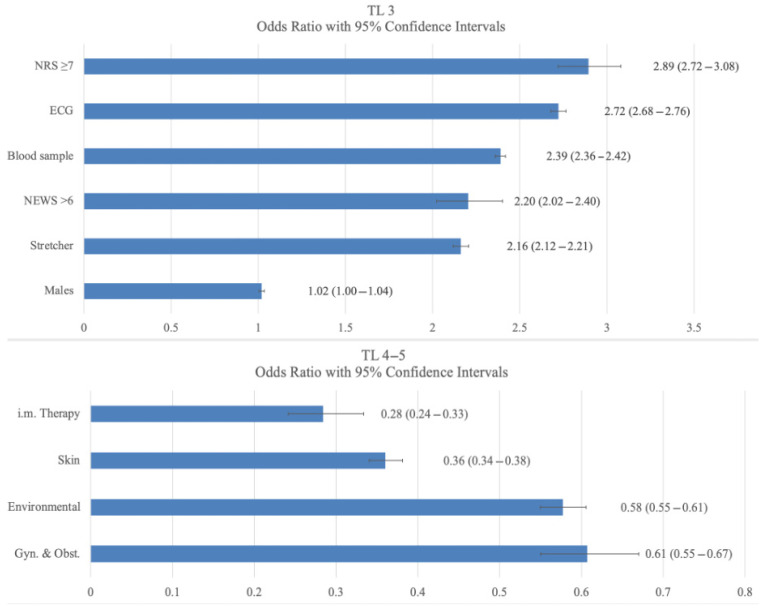
Main features of the PP and CDW, in order of relevance, selected by the logistic model associated with TL3 (**upper panel**) and TL4–5 (**lower panel**). Data are reported as number of cases (%), odds ratio (OR) with 95% confidence intervals (CI). Statistical significance set at *p* < 0.05.

Figure 2 lists the variables selected by the stepwise procedure of the logistic model for predicting the TL3 group (upper panel). The main features were, in order of importance, as follows: NRS > 7, electrocardiogram, blood sampling, NEWS > 6, staying on a stretcher, and male gender. For the TL4–5 group (lower panel), the variables selected in order of importance were the categories of gynaecological–obstetric, environmental and skin symptoms, and intramuscular therapy.

**Figure 3:**
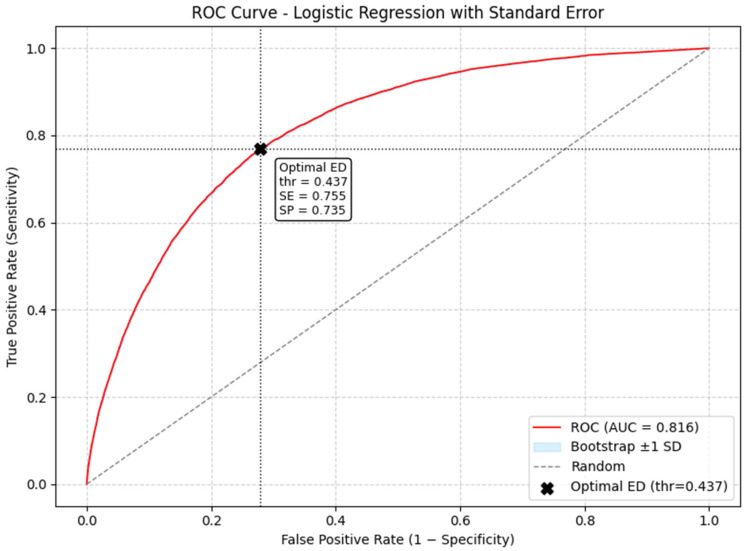
ROC plots of the risk score obtained from the logistic model. Accuracy was 0.816, with sensitivity and specificity at the optimal cutoff point (score of 0.437).

The ROC curve for the risk score is shown in Figure 3. The accuracy in identifying the right TLs was 0.816, with a sensitivity of 0.755 and a specificity of 0.735 at the optimal cutoff point (score of 0.437), simultaneously maximising both sensitivity and specificit

## 4. Discussion

### 4.1. Key Results

This study identified the characteristics of the PP and CDW associated with an HLC in TL3 subjects and LLC in TL4–5 subjects, which are required to complete the ED clinical diagnostic pathway. Namely, severe pain (NRS > 7), requirement for an ECG, requirement for blood sampling for urgent tests, elevated vital sign risk scores (NEWS > 6), the need for placement on a stretcher, and male gender are associated with HLC features. Disorders related to obstetrics and gynaecology, environmental conditions, dermatology, and intramuscular drug therapy were associated with an LLC. 

This study shed light on the current criteria based on the number of resources required, identifying additional characteristics that distinguish between high and low complexity in non-critical cases. Unfortunately, these results cannot be used for a new classification for subjects arriving at triage, as they require both internal and external validation studies [15]. However, these findings provide valuable insights into the current level assignment policy and could inform strategies to enhance the appropriateness of ED care pathways.

In our model, high-severity pain (NRS > 7) at presentation was the main contributor of HLC-associated characteristics, with an increased odds ratio ranging from 2.7 to 3.0. European epidemiological data on chronic and acute pain report that approximately two-thirds of subjects experience moderate pain (NRS 5–7) and only one-third experience severe pain (NRS 8–10) [16]. However, these results may be underestimated, as reported in a Finnish study showing that pain was reported in only 28% of all patients [17].

European guidelines highlight the widespread use of pain assessment in EDs [18], but few data are available on the frequency of cases in the general population and the number of cases referred to the ED. It seems that professionals put more importance on monitoring vital signs, like checking blood pressure or oxygen levels, than on detecting and treating severe pain [19]. In our series, pain on presentation was assessed in 85% of cases. Moderate pain (NRS 4–7) was found in 13.5% of cases and severe pain in only 1.5%. This low percentage could be due to incorrect case detection or the exclusion of critical cases assigned to the very urgent characteristics. Finally, although pain is a key criterion for assignment to the HLC group, our model identifies even a small proportion of cases as the most important factor.

An ECG is a diagnostic test commonly performed in the ED in cases of suspected acute cardiovascular disease, as well as in many other clinical settings [20]. It is estimated that approximately 22–35 out of every 100 patients in the ED undergo an ECG. A 2019 survey of 1000 US EDs found that 28% of patients underwent an ECG [21]. The survey also reported that ECG use has increased by 1% per year over the last 3 years, reflecting an increase in patient complexity [21]. In our series, an ECG was performed on 32.2% of participants, representing the second predictive variable associated with TL3, with an odds ratio of 2.76.

Blood tests in the ED are important for diagnosis and treatment. They are used in triage before medical examination as part of clinical guidelines [22]. The usefulness of blood tests in the ED depends on patient age, severity and length of stay [20,23], but this can lead to an excessive number of tests [24]. In our data, blood tests were taken in 56.8% of cases, with a higher frequency (60.2%) in the TL3 group, in line with the Osman study [22] in which 62% of adult patients classified as less severe underwent blood sampling for laboratory tests.

Early warning scores are useful in identifying clinical deterioration in high-risk patients [25] and can facilitate decision-making when assigning severity codes and allocating resources [26]. Indeed, studies have shown that early warning scores provide additional information for assessing the risk of hospitalisation and mortality compared to traditional triage scales based on patient characteristics, even in non-critical cases [25]. As our study focused on non-critical cases, the NHS Early Warning Score (NEWS) was categorised as mild in 96.0% of cases and moderate in only 2.9%. The NEWS was higher in all categories of the HLC group, despite the low grade. Notwithstanding the limited number of non-critical cases with an NEWS >6, this was one of the most significant variables selected by the statistical model in predicting TL3 characteristics.

The decision whether or not to place a patient on a stretcher is important because it is one of the six main variables selected by the logistic model for identifying TL3. This helps to prioritise bed and staff assignments, optimising patient flow and reducing overcrowding delays. This lends weight to the use of stretcher occupancy as a key metric in resource allocation models and ED operational planning [27]. In our series, 17.7% of non-critical patients (59,311 cases) were assigned to stretcher allocation. This percentage was higher in the TL3 group (26.5%) than in the TL4–5 group (10.9%). Whether or not to place a patient on a stretcher is an important decision because it is one of the six main variables selected by the logistic model for predicting TL3.

Male patients visiting the ED, particularly those in older age categories, have more comorbidities [28] than females and are at greater risk of being admitted to intensive care and of dying. Our study confirms an association between male gender and an HLC on arrival at the ED, albeit with borderline statistical significance. Even in the presence of a statistical result, the clinical significance of the increased risk in males does not appear to be clinically relevant.

### 4.2. Limitations

Several limitations needed to be acknowledged.

First, the study identifies characteristics of non-critical cases, thereby expanding upon the original criteria, which only considered the number of needed resources. The statistical model, which was obtained using real-world data, successfully identifies the relevant variables. However, although this was a multicentre study, it should be noted that these results may not be generalisable due to their dependence on local contexts, even though individual hospitals were not included in the logistic model.

Second, the CEDIS-compliant list was used to classify the main complaints and case-mix. However, the reliability of this classification is questionable, as no analysis of intra- and inter-operator variability has been performed.

Third, the low frequency of procedures associated with cases with minor triage codes due to local organisational characteristics may have led the statistical model to prioritise some typologies to the detriment of other, less represented ones. It should also be considered that the differing representativeness of the variables could produce a statistically significant result with a negligible clinical significance. Examples include cases in the LLC group, obstetric and gynaecological disorders, and environmental disorders.

Fourth, the model showed an accuracy of 0.801 (0.011), with an optimal cutoff point of 0.517. This level of discriminatory power is considered acceptable for clinical purposes [29]. Unfortunately, the model was constructed with the available variables, but these do not take into account the judgement that may have prevailed over traditional variables. These models could be improved by refining the choice of variables, using appropriate outcome measures, analysing the results and eliminating the “self-fulfilling prophecy” effect [29]. Additional variables, such as severity scores, could improve the model’s performance [30].

Fifth, we did not consider subjects who underwent lab tests at triage vs. after the medical exam. This may have increased the number of urgent lab tests, overestimating the effect in the logistic model. We believe early lab test results did not influence the triage level code.

Sixth, there may be a correlation between the PP and their CDW variables in completing the treatment of patients. We do not propose a new classification tool, as this would require internal and external validation before it could be implemented.

Seventh, we considered the EDLoS as one of the variables to be tested in the model, using the actual time interval from arrival to discharge rather than an estimate made by triage nurses at the time of triage. The value obtained a posteriori constitutes a methodological limitation in the interpretation of the results, which must be taken into consideration.

### 4.3. Interpretation

Real-world data from a large study population indicate that certain characteristics of the presentation profile and clinical diagnostic workload are associated with high or low complexity among ED attendees. A stratification criterion based on the PP and CDW could improve the original model, which is based on estimating the number of resources needed to complete the clinical diagnostic pathway. These results pave the way for an in-depth discussion on the level assignment policy currently in use. If confirmed by further studies, following a procedure of both internal and external validation, our results could redefine the criteria for classifying non-critical cases at ED triage. To achieve this goal, additional information not considered by current models may be attained, such as multidimensional assessments in elderly subjects [30], or information on the expected level of care required.

### 4.4. Generalizability

Although the sample analysed was large, the study was conducted in an area with a homogeneous organisational model. Therefore, we cannot rule out the possibility that the results may not be fully replicable in areas with different organisational models.

## Figures and Tables

**Table 1:** Baseline characteristics of the two groups. Data presented as number (%), with odds ratios (OR) and 95% confidence intervals (CI). Statistical significance set at *p* < 0.05. Age reported as median with [interquartile range].

	Total, No.	TL3, No. (%)	TL4–5, No. (%)	OR (95% CI)	*p*-Value
**Patients**	335,507	145,355 (43,3)	190,152 (56.7)	--	--
**Sex** (Male)	168,457 (50.2)	71,352 (49.1)	97,105 (51.1)	1.08 (1.07–1.10)	<0.001
**Age** (Years)	59 (interquartile range [IQR], 25)	69 (interquartile range [IQR], 32)	54 (interquartile range [IQR], 34)	--	<0.05
18–30	39,590 (11.8)	10,866 (7.5)	28,724 (15.1)	2.20 (2.15–2.25)	<0.001
31–40	35,080 (10.5)	10,593 (7.3)	24,487 (12.9)	1.88 (1.84–1.93)	<0.001
41–50	42,180 (12.6)	14,012 (9.6)	28,168 (14.8)	1.63 (1.60–1.67)	<0.001
51–60	50,966 (15.2)	18,743 (12.9)	32,223 (16.9)	1.38 (1.35–1.41)	<0.001
61–70	45,020 (13.4)	19,811 (13.6)	25,209 (13.3)	0.97 (0.95–0.99)	0.002
70+	122,671 (36.6)	71,330 (49.1)	51,341 (27.0)	0.38 (0.38–0.39)	<0.001
**NEWS**					
0 to 4	322,409 (96.1)	136,608 (94)	185,801 (97.7)	2.73 (2.63–2.84)	<0.001
5 to 6	9579 (2.9)	6078 (4.2)	3501 (1.8)	0.43 (0.41–0.45)	<0.001
>6	3519 (1.0)	2669 (1.8)	850 (0.4)	0.24 (0.22–0.26)	<0.001
**NRS**					
0 to 3	285,197 (85.0)	120,474 (82.9)	164,723 (86.6)	1.34 (1.31–1.36)	<0.001
4 to 7	45,273 (13.5)	21,809 (15.0)	23,464 (12.3)	0.80 (0.78–0.81)	<0.001
>7	5037 (1.5)	3072 (2.1)	1965 (1.0)	0.48 (0.46–0.51)	<0.001

**Table 2:** Chief complaint categories in subjects attending the ED. Data presented as number of cases (%) with odds ratio (OR) and 95% confidence intervals (95% CI). Statistical significance set at *p* < 0.05.

Type	Total No. (%)	TL3 No. (%)	TL4–5 No. (%)	OR (95% CI)	*p*-Value
Environmental (ENV)	283 (0.1)	168 (0.1)	115 (0.1)	2.88 (2.25–3.68)	<0.001
Mental Health (MH0)	7172 (2.1)	3369 (2.3)	3803 (2.0)	2.03 (1.93–2.14)	<0.001
Cardiovascular System (CVS)	54,412 (16.2)	38,772 (26.7)	15,640 (8.2)	1.94 (1.88–2.00)	<0.001
Cerebral Nervous System (CNS)	32,520 (9.7)	17,905 (12.3)	14,615 (7.7)	1.64 (1.59–1.69)	<0.001
Trauma (TRA)	14,888 (4.4)	6484 (4.5)	8404 (4.4)	1.56 (1.49–1.62)	<0.001
Respiratory (RES)	21,747 (6.5)	13,839 (9.5)	7908 (4.2)	1.44 (1.39–1.50)	<0.001
Substance Misuse (SUB)	1366 (0.4)	577 (0.4)	789 (0.4)	1.40 (1.25–1.57)	<0.001
Genitourinary (GU0)	24,477 (7.3)	10,917 (7.5)	13,560 (7.1)	0.97 (0.94–1.01)	0.171
Gastrointestinal (GI0)	46,318 (13.8)	22,866 (15.7)	23,452 (12.3)	0.91 (0.88–0.94)	<0.001
General and Minor (GEN)	27,684 (8.2)	11,958 (8.2)	15,726 (8.3)	0.73 (0.70–0.75)	<0.001
Obstetrics/Gynaecology (GYN)	2688 (0.8)	569 (0.4)	2119 (1.1)	0.70 (0.64–0.77)	<0.001
Orthopaedic (ORT)	69,901 (20.8)	13,068 (9.0)	56,833 (29.9)	0.48 (0.46–0.49)	<0.001
Ears, Mouth, Throat, Neck, Nose (ENT)	11,792 (3.5)	2391 (1.6)	9401 (4.9)	0.47 (0.45–0.50)	<0.001
Ophthalmology (OPT)	7939 (2.4)	894 (0.6)	7045 (3.7)	0.44 (0.41–0.48)	<0.001
Dermatology (DER)	12,319 (3.7)	1578 (1.1)	10,741 (5.6)	0.36 (0.34–0.38)	<0.001

## Data Availability

The data presented in this study are available on request from the corresponding author.

## Supplementary Materials

The following supporting information can be downloaded at: https://www.mdpi.com/article/10.3390/jcm15031280/s1, Table S1: List of laboratory tests and interventions included in the CDW considered for the analyses in patients attending the ED with TL3 and TL4–5; Table S2: Number of resources needed in TL3 and TL4–5 groups; Figure S1: The distribution of decades in relation to gender in the TL3 and TL4–5 groups.

## Abbreviations

The following abbreviations are used in this manuscript:

CCI	Charlson Comorbidity Index
CDW	Clinical diagnostic workload
CHF	Congestive Heart Failure
CKD	Hemiplegia, Chronic Kidney Disease
COPD	Chronic Obstructive Pulmonary Disease
CVA	Acute Cerebrovascular Accidents or Transient Ischemic Attacks
D	Dementia
DM	Diabetes Mellitus
ED	Emergency Department
GTD	Connective Tissue diseases,
HLC	High Complexity Level
L	Lymphoma and Leukaemia
LD	Liver Disease
LLC	Low Level of Complexity
MI	History of Myocardial Infarction
NEWS	National Early Warning Score
NRS	Numeric rating scale
PP	Presenting Profile
PUD	Peptic Ulcer Disease
PVD	Peripheral Vascular Disease
ROC	Receiver Operating Characteristic
ST	Solid Tumours
TL	Triage level
