# Supplementary material for: High- and Low-Complexity Features of Non-Critical Adult Patients in the Emergency Department
Source: J Clin Med. 2026 Feb 5;15(3):1280. doi: 10.3390/jcm15031280 (PMC12897838; doi:10.3390/jcm15031280)
Supplement: Supplementary file 1 [file jcm-15-01280-s001.zip › Supplementary Table S2 .pdf]

Table 2A

Number of resources needed in relation to triage level (TL) 3 and TL4-5 in subjects attending the ED.

	**Total, No.**	**TL 3, No. (%)**	**TL 4-5, No. (%)**	**OR (95% CI)**	**-value**
**Patients**	335,507	145,355 (43,3)	190,152 (56.7)	--	--
0 Resource	06.017 (31.6)	17.234 (11.9)	88.783 (46.69)	0.00 (0.00 – 0.00)	0.000
1 Resource	77.750 (23.2)	31.948 (22.0)	45.802 (24.09)	0.00 (0.00 – 0.00)	0.000
2 Resources	75.801 (22.6)	44.208 (30.4)	31.593 (16.61)	0.00 (0.00 – 0.00)	0.000
≥3 Resources	75.938 (22.6)	51.965 (35.7)	23.973 (12.61)	0.00 (0.00 – 0.00)	0.000
