# Supplementary material for: High- and Low-Complexity Features of Non-Critical Adult Patients in the Emergency Department
Source: J Clin Med. 2026 Feb 5;15(3):1280. doi: 10.3390/jcm15031280 (PMC12897838; doi:10.3390/jcm15031280)
Supplement: Supplementary file 1 [file jcm-15-01280-s001.zip › Supplementary Table S1.pdf]

Table A1: List of tests and interventions included in the clinical diagnostic workload considered for the analyses in patients attending the ED with TL3 and TL4-5.

Features	N. =	%	Features	N. =	%
Blood sample	240,385	71,7%	Aerosol Therapy	262	0,1%
E.C.G.	117,754	35,1%	Swab Tests	234	0,1%
Peripheral i.v. Cath.	40,325	12,0%	I.V therapy	204	0,1%
Swab Cov2 Test	40,128	12,0%	Foreign Body Removal	207	0,1%
Specialist Advice	24,211	7,2%	Arthrocentesis	152	0,1%
Urine Dipstick	14,092	4,2%	Ocular Medication	158	0,1%
Complex Medication	7,281	2,2%	Bone Fracture Reduction	119	0,0%
ECHO	7,088	2,1%	E.C.G. Consult	84	0,0%
Vitals Monitoring	2,978	0,9%	Skin Incision	79	0,0%
Suture	2,321	0,7%	Blood Transfusion	65	0,0%
I.M. Therapy	1,680	0,5%	Compressive Ultra Sonography	42	0,0%
C-Spine Immob.	1,533	0,5%	Non Invasive Ventilation	37	0,0%
Bandage	980	0,3%	Rachicenthesis	46	0,0%
Immune Therapy	861	0,3%	Cardio-Pulmonary Resuscitation	30	0,0%
Local Anesthesia	803	0,2%	Thoracentesis	14	0,0 %
Capillary Blood Sample	681	0,2%	Central Venous Catheter	7	0,0 %
Naso-Gastric Tube	458	0,1%	Paracentesis	5	0,0 %
