# Supplementary figures and images for: High- and Low-Complexity Features of Non-Critical Adult Patients in the Emergency Department
Source: J Clin Med. 2026 Feb 5;15(3):1280. doi: 10.3390/jcm15031280 (PMC12897838; doi:10.3390/jcm15031280)

Figure A1

Age Distributions by Triage Level and Gender

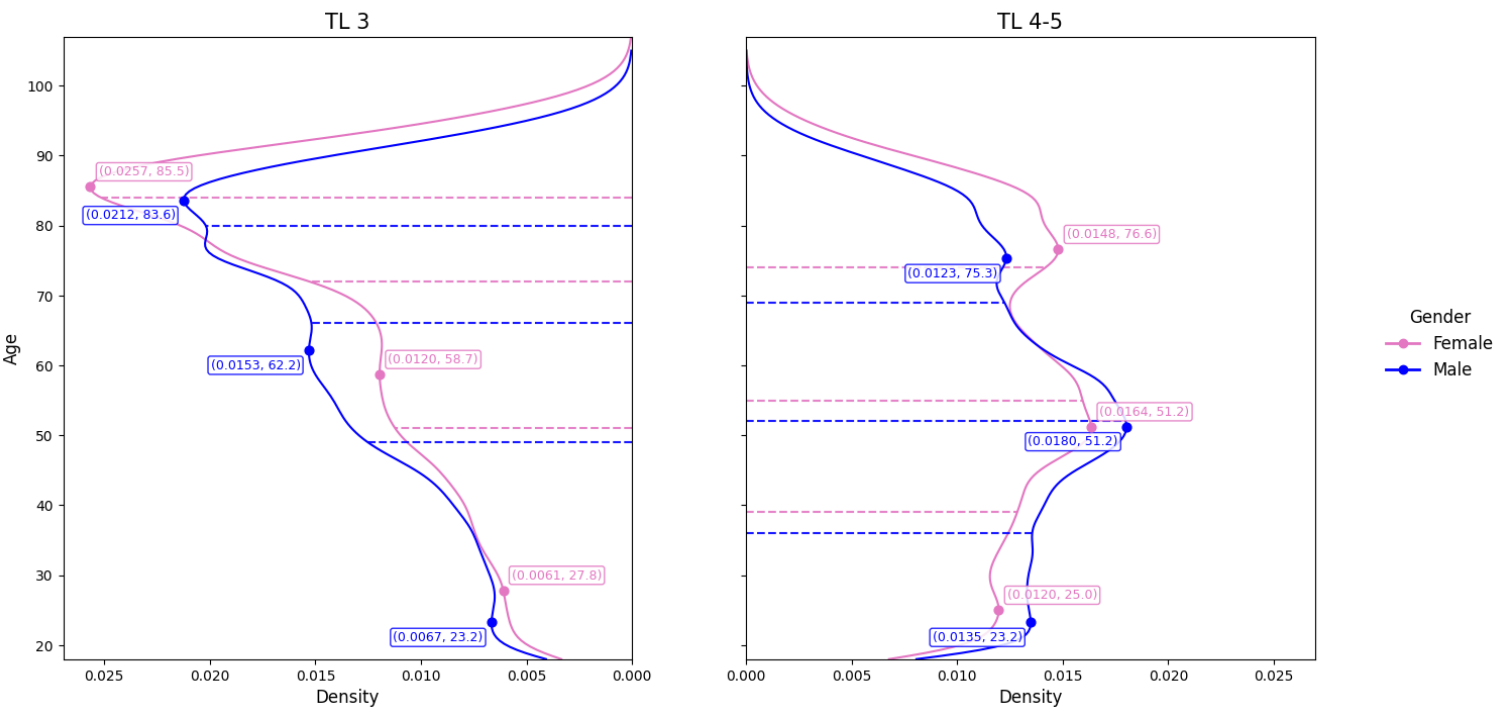

Supplement: Supplementary file 1 [file jcm-15-01280-s001.zip › Supplementary Figure S1.pdf]
